# Visual Cognitive Assessment Test: Utility of the brief cognitive battery for early screening of cognitive impairment in Chongqing, China

**DOI:** 10.1002/brb3.3413

**Published:** 2024-02-05

**Authors:** Yidan Liu, Binbin Xie, Qin Li, Shufang Xiao, Jiamin Li, Nagaendran Kandiah, Kok Pin Ng, Liumi Jiang, Xiaofeng Li

**Affiliations:** ^1^ Department of Neurology the Second Affiliated Hospital of Chongqing Medical University Chongqing China; ^2^ Department of Neurology Chengdu seventh People's Hospital Chengdu China; ^3^ Department of Neurology National Neuroscience Institute Singapore Singapore; ^4^ Duke‐NUS Medical School Singapore Singapore; ^5^ Lee Kong Chian School of Medicine Nanyang Technological University Singapore Singapore Singapore; ^6^ Department of Neurology the Ninth People's Hospital of Chongqing Chongqing China

**Keywords:** cognitive impairment, diagnostic value, screening test, the Visual Cognitive Assessment Test (VCAT), reliability

## Abstract

**Objectives:**

Early detection of cognitive impairment is essential for timely intervention. Currently, most widely used cognitive screening tests are influenced by language and cultural differences; therefore, there is a need for the development of a language‐neutral, visual‐based cognitive assessment tool. The Visual Cognitive Assessment Test (VCAT), a 30‐point test that assesses memory, executive function, visuospatial function, attention, and language, has demonstrated its utility in a multilingual population. In this study, we evaluated the reliability, validity, and diagnostic performance of the VCAT for screening early cognitive impairment in Chongqing, China

**Methods:**

A total of 134 individuals (49 healthy controls (HCs), 52 with mild cognitive impairment (MCI), and 33 with mild dementia) completed the Mini‐Mental State Examination (MMSE), Montreal Cognitive Assessment (MoCA), VCAT, and domain‐specific neuropsychological assessments. The diagnostic performances of MMSE, MoCA, and VCAT were evaluated using the area under the curve (AUC), sensitivity, and specificity. Construct validity of the VCAT was assessed with well‐established domain‐specific cognitive assessments. Reliability was measured using Cronbach's alpha.

**Results:**

The VCAT and its subdomains demonstrated both good construct validity and internal consistency (α = 0.577). The performance of VCAT was comparable to that of MoCA and MMSE in differentiating mild dementia from nondemented groups (AUC: 0.940 vs. 0.902 and 0.977, respectively; *p* = .098 and .053) and in distinguishing cognitive impairment (CI) from HC (AUC: 0.929 vs. 0.899 and 0.891, respectively; *p* = .239 and .161), adjusted for education level. The optimal score range for VCAT in determining dementia, MCI, and HC was 0–14, 15–19, and 20–30, respectively.

**Conclusion:**

The VCAT proves to be a reliable screening test for early cognitive impairment within our cohort. Being both language and cultural neutral, the VCAT has the potential to be utilized among a wider population within China.

## Graphical abstract

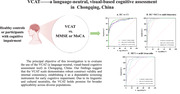

## 1 INTRODUCTION

With the global aging population, the prevalence of dementia has rapidly increased, emerging as a significant global public health concern (Ferri et al., 2005; Prince et al., 2013). Given the escalating burden of cognitive impairment, early identification becomes crucial for subsequent intervention and treatment. Mild cognitive impairment (MCI) is a condition characterized by cognitive impairment with minimal impact on instrumental activities of daily living (Tangalos & Petersen, 2018). A random‐effects meta‐analysis revealed that, over two years, the cumulative incidence of individuals with MCI aged over 65 developing dementia was 14.9% (Petersen et al., 2018). Therefore, MCI represents a critical window for early diagnosis and timely intervention. The diagnosis of both MCI and mild dementia necessitates a comprehensive approach, including a detailed clinical history, neuropsychological assessment, relevant laboratory investigations, and neuroimaging. However, such a thorough evaluation is both time‐consuming (Chertkow et al., 2013) and impractical in a busy clinical practice with a large cohort of older individuals. Hence, having a brief yet accurate cognitive screening test for detecting early cognitive impairment will significantly aid clinicians in managing large clinic cohorts (Perneczky, 2019). Moreover, timely identification of cognitive impairment empowers individuals to make pivotal decisions concerning the orchestration of their forthcoming existence, encompassing adept care strategies and interventions (Cohen‐Mansfield, 2005; Perneczky, 2019). However, cognitive screening may also engender psychological burdens, necessitating prompt psychological guidance.

The most commonly used screening tests worldwide for detecting MCI and dementia are the Montreal Cognitive Assessment (MoCA) and the Mini‐Mental State Examination (MMSE), respectively (Folstein et al., 1975; Tang, 2020). However, these tests necessitate translation, adaptation, and validation before being applied in populations with different linguistic or cultural backgrounds (Bender et al., 2010). During this process, the neuropsychological foundation of specific test items is susceptible to alteration. For example, in countries with languages lacking syllables, alphabet fluency is often replaced by category fluency. This substitution results in the measurement of different psychometric attributes in various language groups, hindering meaningful cross‐lingual and cross‐cultural comparisons in international collaborations and clinical trials (Bender et al., 2010). The Visual Cognitive Assessment Test (VCAT) scale, a cross‐cultural, language‐neutral test, was developed within this specific context in Singapore (Kandiah et al., 2016; Koh et al., 2020; Lim et al., 2018).

In Chongqing, China, the elderly population, comprising the primary cohort for cognitive function assessment, exhibits a diminished educational level, and some struggle with reading. This may affect the outcomes of text‐based cognitive screening tools. Differing from the MMSE and MoCA tests, the VCAT exclusively utilizes visual representations, eliminating the need for textual elements (Gauthier et al., 2021). The image‐based assessment tools are highly intuitive, potentially offering convenience for evaluators and greater acceptance among individuals with limited education, as compared to traditional textual questionnaires. Therefore, the VCAT may be exceptionally well suited for cognitive screening in Chongqing, China. This study aimed to evaluate the reliability and validity, along with the ease of implementation and level of acceptance by both evaluators and the subjects under evaluation, of the VCAT test within the healthcare system of Chongqing, China.

## 2 MATERIALS AND METHODS

### 2.1 Study participants

A total of 134 participants sharing a common Chinese language and cultural background were recruited, including 49 healthy controls (HC), 52 patients with MCI, and 33 patients with mild dementia. The recruitment took place at the Memory Clinic and inpatient department of the Second Affiliated Hospital of Chongqing Medical University from June 2019 to July 2021. All study participants, aged between 52 and 97, with adequate visual and auditory acuity, completed the study protocol, which included cognitive testing, laboratory screening (vitamin B12, folic acid, thyroid hormone levels, rapid plasma regain (RPR), and treponema pallidum particle agglutination (TPPA)), and cranial CT/MRI scans to exclude clinically significant abnormalities. Participants with severe neurological disease, psychiatric disorders/psychotic features, severe visual impairment, and depression (Geriatric Depression Scale (GDS) (Smarr & Keefer, 2011) scored more than 10 in the preceding 2 weeks) were excluded. The classification of HC, MCI, and mild dementia was determined by neurologists based on a thorough review of medical history, neuropsychological assessment (Salmon & Bondi, 2009), and neuroimaging.

The inclusion criteria for the HC group were as follows: no memory complaints or difficulties, normal cognitive performance, and a Clinical Dementia Rating (CDR) score of 0 (Morris, 1993). MCI was diagnosed using the Peterson criteria (Petersen, 2004), which include memory complaints and difficulties verified by an informant, symptoms lasting more than 3 months, preserved basic activities of daily living (ADL) or minimal impairment in complex instrumental functions, and not meeting diagnostic criteria for dementia according to the National Institute on Aging‐Alzheimer's Association (NIA‐AA) working group (McKhann et al., 2011). Mild dementia was diagnosed based on the absence of significant impairment in cognitive functions or ADL, meeting the criteria for probable dementia according to the NIA‐AA working group, and a CDR score of 1. Ethical approval was obtained from the Ethical Committee of the Second Affiliated Hospital of Chongqing Medical University, and informed consent was obtained from all participants.

### 2.2 Neuropsychological assessments

Participants were administered a standardized battery of neuropsychological assessments (NPA) in a private and quiet environment by trained neurologists (Salmon & Bondi, 2009). The NPA comprised the VCAT scale and other established tests to measure global cognition. The VCAT scale constitutes a scoring framework derived from effortlessly and accurately identified images, evaluating memory, executive function, visual‐spatial acumen, attention, and language, yielding a composite score of 30 points. In the memory domain, VCAT assesses immediate and delayed recall of visual scenes, as well as the recall of shapes and objects. Executive function tasks involve patients in identifying mechanisms related to gear rotation, pattern recognition, and picture categorization. The language domain includes a category fluency task and an image naming task. Visual‐spatial tasks evaluate patients' capacity to reconstruct space and navigate grids. Lastly, the attention domain encompasses a shape elimination task. Specific details of the VCAT scale were provided in Supplemental Material 1–3.

Global cognition was assessed using the MMSE and the Beijing adaptation of the MoCA. Episodic memory function was measured through the 15‐Word Immediate Memory (IM), Delayed Memory (DM), and Cued Memory Task (CM). Attention was evaluated using the Digit Span Forward Test (DSFT) (Katsoulaki et al., 2017). Executive function was assessed with the Digit Span Backward Test (DSBT) (Katsoulaki et al., 2017) and Trail making Test (TMT‐A and TMT‐B) (Guo, 2022). Visuospatial abilities were examined using the Clock Drawing Test (CDT) (Shulman, 2000). Language was evaluated using the Boston Naming Test (BNT) (Sachs et al., 2020). All tests demonstrated good reliability and validity within the Chinese cultural context (Guo et al., 2012). We sequentially conducted assessments for each patient using VCAT, MMSE, MoCA, IM, DFST, DSBT, TMT‐partA, TMT‐partB, CDT, BNT, DM, and CM. The intervals between each scale were approximately 1 min, during which we engaged in brief conversations with the patients and provided guidance for the next assessment. A total of three neurologists, all undergoing specialized training in scale assessments, participated in the evaluations.

### 2.3 Statistical analysis

Statistical analysis was performed using SPSS (Version 20.0) and MedCalc. Continuous test variables were assessed for normality using the Shapiro–Wilk test. One‐way analysis of variance (ANOVA) was used to perform between‐group comparisons on normally distributed continuous data and post hoc pairwise comparisons between groups were assessed using the least significant difference (LSD) test. The continuous data with skewed distribution were analyzed using the Kruskal–Walli's test. Categorical data were analyzed using Chi‐square tests of independence. The Cronbach's alpha was used to measure the internal consistency. Spearman's correlation test was used to evaluate the association between cognitive tests and cognitive domain of VCAT. Multivariate linear regression models were utilized to investigate the influence of education years, and age on VCAT, MMSE, and MoCA. Receiver operating characteristic (ROC) analysis was used to assess the sensitivity, specificity, and cutoff values for MMSE, MoCA and the VCAT. Optimal cutoff scores were defined as maximal sensitivity and specificity, measured by Youden's Index. The area under the ROC curves (AUC) was used as an overall index of performance of the screening tests; the AUCs and their standard errors were calculated using the method of Hanley and McNeil. The efficacy of the combined diagnosis of the MMSE/MoCA and VCAT was examined using logistic regression and ROCs. Subgroup analysis for participants with limited educational attainment (< 9 years of education) was conducted to elucidate the efficacy of VCAT in lower education population. Two‐tailed *p* value of .05 was considered statistically significant for all tests.

## 3 RESULTS

### 3.1 Baseline characteristics

Baseline demographics and neuropsychological assessment scores of the three groups were presented in Table 1. The VCAT scores were recorded as 24.08 ± 3.62 for the HC group, 17.15 ± 4.25 for the MCI group, and 10.61 ± 3.35 for the mild dementia group. Significant age differences were observed when comparing MCI or mild dementia to HC (*p* < .01). However, there were no significant differences in sex and education among the three groups (*p* > .05). As expected, we identified significant differences in global and subdomain cognitive scores between HC, MCI, and mild dementia, confirming the appropriate stratification of subjects into their respective groups. The average time required to administer the VCAT in our cohort was 852.17 ± 117.48 seconds (ranging from 12 to 16 min). The Cronbach's alpha of the VCAT was .577, indicating a satisfactory level of reliability.

**TABLE 1:** Demographics and standardized neuropsychological tests for the three groups.

	HCs mean (SD) (*n* = 51)	MCI mean (SD) (*n* = 35)	Mild dementia Mean (SD) (*n* = 21)	*p* Value
MMSE^b^ (*M* = 30)	27.69 (1.78)	24.44 (2.95)**	16.55 (3.70) ##$$	<.001
MoCA^b^ (*M* = 30)	23.35 (2.63)	18.35 (4.31)**	12.73 (4.19) ##$$	<.001
VCAT^b^ (*M* = 30)	24.08 (3.62)	17.15 (4.25)**	10.61 (3.35) ##$$	<.001
IM^b^ (*M* = 45)	21.16 (3.89)	15.94 (3.94)**	10.82 (4.47) ##$$	<.001
DFST^b^ (*M* = 12)	7.69 (1.54)	7.52 (1.50)	6.70 (1.55) #$$	.006
DSBT^b^ (*M* = 12)	4.08 (1.34)	3.58 (1.05)	3.27 (1.04) $$	.009
TMT‐partA^b^ (s)	53.43 (24.32)	89.04 (46.21)**	110.36 (40.63) #$$	<.001
TMT‐partB^b^ (s)	95.59 (55.86)	180.50 (88.56)**	236.82 (87.65) ##$$	<.001
CDT^b^ (*M* = 3)	2.82 (0.49)	2.37 (0.89)**	1.55 (0.97) ##$$	<.001
BNT^b^ (*M* = 30)	24.08 (3.11)	20.35 (4.90)**	18.27 (4.65) #$$	<.001
DM^b^ (*M* = 15)	6.57 (2.79)	2.13 (2.59)**	0.70 (1.26) #$$	<.001
CM^b^ (*M* = 12)	8.92 (2.44)	4.17 (2.79)**	1.21 (1.69) ##$$	<.001
Time^c^ (min)	13.57 (2.02)	14.36 (1.69) **	14.89 (2.03) $	.008
Age^c^ (years)	69.12 (7.87)	73.12 (7.66) **	73.85 (6.81) $$	.008
Edu^b^ (years)	10.90 (3.56)	10.54 (5.16)	10.48 (4.21)	.859
Sex^a^ (F/M)	32:17	32:20	22:11	.872

*Note*: Comparison between HCs group and MCI group is marked behind “MCI group”; *p < .05; **p < .01. Comparison between MCI group and mild dementia group is marked behind “mild dementia group”; #*p <* .05; ##*p <* .01. Comparison between HCs group and mild dementia group is marked behind “mild dementia”; $*p <* .05; $$*p <* .01.

^a^ Chi‐square test, *M* = maximum.

^b^ Mann–Whitney *U*.

^c^ ANOVA.

HCs, healthy control; MCI, mild cognitive impairment; MMSE, Mini‐Mental State Examination; MoCA, Montreal Cognitive Assessment; VCAT, Visual Cognitive Assessment Test; IM, immediate memory; DFST, the Digit Span Forward Test; DSBT, the Digit Span Backward; TMT, Trail Making Test; CDT, Clock Drawing Test; BNT, Boston Naming Test; DM, delay memory; CM, clue‐memory; SD, standard deviation.

### 3.2 Influence of age and education levels on VCAT

Multivariate linear regression models were employed to assess the impact of participant characteristics on VCAT, MMSE, and MoCA scores within the current cohort. Age exhibited a modest negative correlation with both VCAT and MoCA, while gender demonstrated no discernible association with any of the three scores. Conversely, education years displayed a significant positive correlation with all three scores. Notably, the effect of education on VCAT (β (95% CI): 0.29 (0.03−0.54)) was less pronounced compared to MMSE (β (95% CI): 0.34 (0.15−0.55)) and MoCA (β (95% CI): 0.48 (0.28−0.69)) (Table S1).

### 3.3 Construct validity

Significant correlations were identified between the VCAT subdomains and their corresponding NPA subdomains, supporting the construct validity of the VCAT (Table 2). The correlation coefficients between VCAT episodic memory and the IM, DM, and CM scores were 0.630, 0.722, and 0.770, respectively (*p* < .01). The correlation coefficient between VCAT attention and TMT‐A was 0.373 (*p* < .01), while the correlation coefficient between VCAT executive function and TMT‐B was 0.483 (*p* < .01). Additionally, the correlation coefficient between VCAT visuospatial and CDT was 0.545 (*p* < .01), and the correlation coefficient between VCAT language and BNT was 0.504 (*p* < .01). In most of the aforementioned analyses, we observed moderate to high correlations, with the exception of DSFT and attention, which showed a lowest correlation (*r* = 0.222, *p* < .05).

**TABLE 2:** Construct validity—correlational matrix comparing VCAT domains against other neuropsychological assessment (NPA) domains.

	VCAT total	Episodic memory	Attention	Executive function	Visuospatial	Language
VCAT		0.898^**^	0.575^**^	0.673^**^	0.390^**^	0.594^**^
MMSE	0.785^**^	0.715^**^	0.382^**^	0.521^**^	0.430^**^	0.520^**^
MoCA	0.795^**^	0.656^**^	0.445^**^	0.590^**^	0.475^**^	0.582^**^
Immediate memory	0.608^**^	0.630^**^	0.180‘	0.279^**^	0.252^*^	0.496^**^
Delay memory	0.652^**^	0.722^**^	0.274^**^	0.253^*^	0.174^*^	0.375^**^
Cued memory	0.757^**^	0.770^**^	0.347^**^	0.407^**^	0.229^*^	0.447^**^
Digit Span Forward Test	0.315^**^	0.210^*^	0.222^*^	0.259^*^	0.336^**^	0.262^*^
Digit Span Backward Test	0.335^**^	0.182^*^	0.271^*^	0.398^**^	0.361^**^	0.220^*^
TMT‐A	−0.565^**^	−0.448^**^	−0.373^**^	−0.412^**^	−0.450^**^	−0.433^**^
TMT‐B	−0.633^**^	−0.502^**^	−0.395^**^	−0.483^**^	−0.498^**^	−0.459^**^
Clock Drawing Test	0.574^**^	0.401^**^	0.436^**^	0.438^**^	0.545^**^	0.498^**^
Boston Naming Test	0.643^**^	0.491^**^	0.402^**^	0.511^**^	0.446^**^	0.504^**^

*Note*: Figures represent Spearman's correlation coefficient. Shaded figures highlight the correspondent VCAT and NPA cognitive domains.

^*^ *p <* .05.

^**^ *p <* .01.

The individual VCAT subdomains demonstrated stronger correlations with their corresponding NPA subdomains compared to other NPA subdomains, except for the VCAT executive domain, which exhibited a stronger association with BNT (*r* = 0.551) than with TMT‐B (*r* = 0.483), and the VCAT attention domain, which showed a stronger association with CDT (*r* = 0.436) than with DSFT (*r* = 0.222). These findings supported the good discriminant validity of the VCAT (Table 2).

The correlation coefficients between the VCAT total score and MMSE were 0.785, and with MoCA were 0.795. Notably, the item with the highest correlation coefficient was episodic memory (*r* = 0.898), while the item with the lowest correlation coefficient was visuospatial (*r* = 0.390) (Table 2).

### 3.4 Optimal VCAT cutoff scores to discriminate NC, MCI, and mild dementia

The optimal thresholds for discerning individuals with MCI were found to be between 15 and 19 total VCAT scores. Scores above 19 suggest healthy individuals, while scores below 15 indicate patients with dementia, as indicated in Table 3.

**TABLE 3:** Receiver operating characteristic (ROC) analysis of the MMSE, the MOCA, and the VCAT.

	Index	ROC area under the curve	95% confidence interval	Cutoff point	Sensitivity (%)	Specificity (%)
HCs vs. MCI + mild dementia	MMSE	0.891	0.826–0.939	26	0.777	0.898
	MoCA	0.899	0.835–0.944	21	0.777	0.857
	VCAT	0.929	0.872–0.966	20	0.847	0.878
	MMSE+ MoCA	0.915	0.855–0.956	–		
	MMSE+ VCAT	0.944	0.890–0.976	–		
HCs + MCI vs. mild dementia	MMSE	0.977	0.935–0.995	21	0.909	0.941
	MoCA	0.902	0.838–0.946	19	0.939	0.743
	VCAT	0.940	0.886–0.947	15	0.909	0.861
	MMSE + MoCA	0.977	0.936–0.995	–		
	MMSE + VCAT	0.979	0.939–0.996	–		
MCI vs. mild dementia	MMSE	0.957	0.889–0.989	21	0.909	0.885
	MoCA	0.821	0.722–0.895	19	0.939	0.539
	VCAT	0.889	0.802–0.947	15	0.909	0.750
	MMSE + MoCA	0.963	0.898–0.992	–		
	MMSE + VCAT	0.961	0.895–0.991	–		

### 3.5 ROC analyses of VCAT, MoCA, and MMSE

**FIGURE 1:**
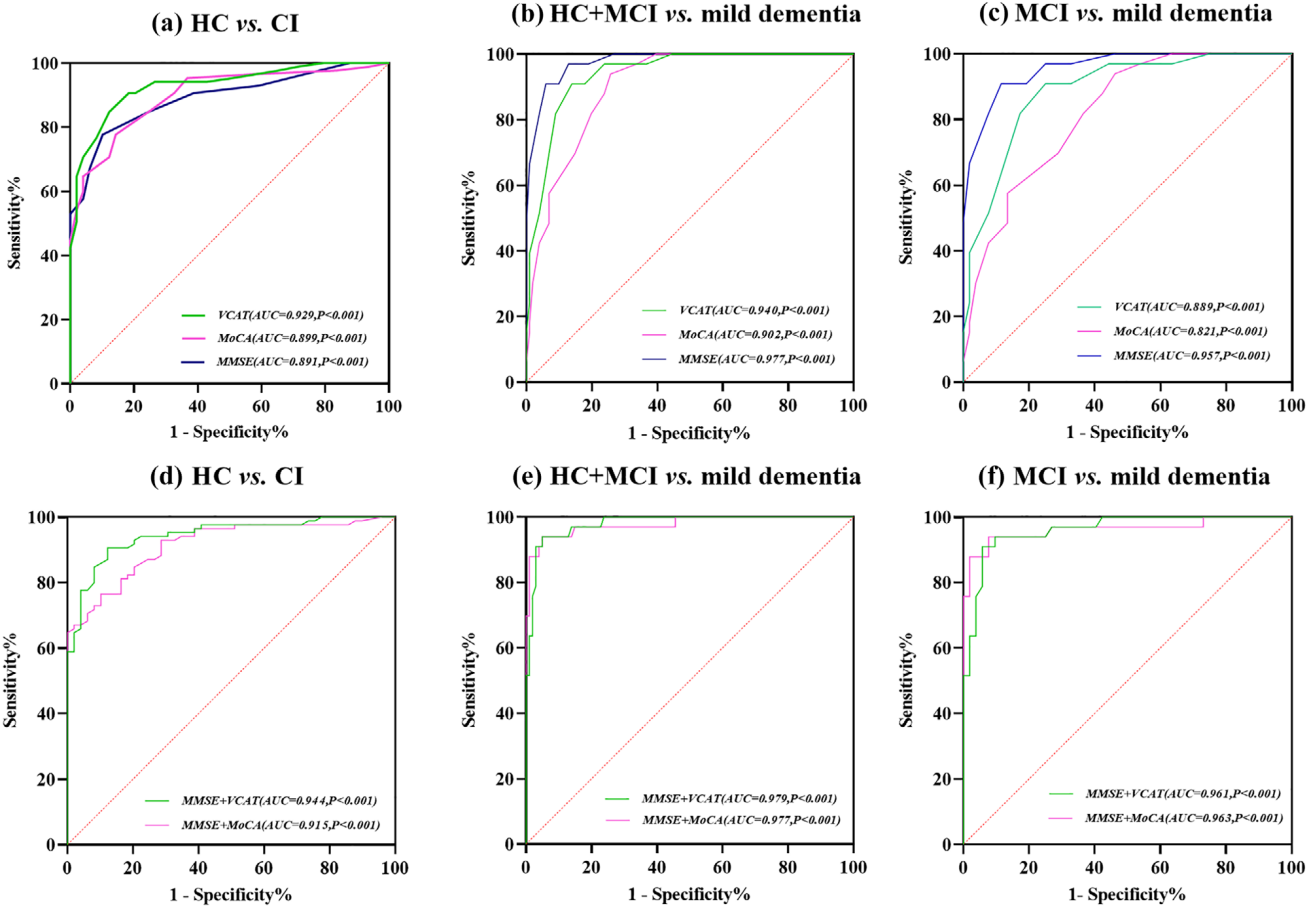
Receiver operating characteristic (ROC) curves of VCAT, MoCA and MMSE. (A, D) ROC curves separating CI (*n* = 56) from HCs (*n* = 51); (B, E) ROC curves separating mild dementia (*n* = 21) from HCs and participants with MCI (*n* = 86); (C, F) ROC curves separating mild dementia (*n* = 21) from MCI (*n* = 35). The diagonal broken line represents the reference line. HCs, healthy controls; CI, cognitive impairment; MCI, mild cognitive impairment; MMSE, Mini‐Mental State Examination; VCAT, Visual Cognitive Assessment Test; MoCA, Montreal Cognitive Assessment; AUC, area under curve.

When discriminating cognitive impairment (MCI + mild dementia) from HC, ROC analyses revealed an AUC of 0.929 (95% CI: 0.872–0.966) for VCAT, with a sensitivity of 0.847 and specificity of 0.878 based on the cutoff value below 20. In comparison, the AUC for MMSE was 0.891 (95% CI: 0.826–0.939), and for MoCA, it was 0.899 (95% CI: 0.835–0.944) (Table 3). No significant difference in the AUC was observed between VCAT and MMSE (*Z* = 1.402, *p* = .161) or between VCAT and MoCA (*Z* = 1.177, *p* = .239) (Figure 1a).

When distinguishing mild dementia from nondemented groups (HC + MCI), ROC analyses yielded an AUC of 0.940 (95% CI: 0.886–0.947) for VCAT, with a sensitivity of 0.909 and specificity of 0.941 based on the cutoff value below 15. In comparison, the AUC for MMSE was 0.977 (95% CI: 0.935–0.995), and for MoCA, it was 0.902 (95% CI: 0.838–0.946) (Table 3). No significant difference in the AUC was observed between VCAT and MMSE (*Z* = 1.937, *p* = .053), or between VCAT and MoCA (*Z* = 1.655, *p* = .098) (Figure 1b).

When differentiating MCI from the mild dementia group, ROC analyses yielded an AUC of 0.889 (95% CI: 0.802–0.947) for VCAT, with a sensitivity of 0.909 and specificity of 0.750 based on the cutoff value below 15. In comparison, the AUC for MMSE was 0.957 (95% CI: 0.889–0.989), and for MoCA, it was 0.821 (95% CI: 0.722–0.895) (Table 3). No significant difference in the AUC was found between VCAT and MoCA (*Z* = 1.555, *p* = .120). However, the AUC of the MMSE score was significantly higher than that of MoCA (*Z* = 3.936, *p* = .001) and VCAT (*Z* = 1.993, *p* = < .05) (Figure 1c). To explore the value of a combination of cognitive tests, ROC analyses indicated that the MMSE and VCAT combination was comparable to the MMSE and MoCA combination in differentiating HC from cognitive impairment participants (*Z* = 1.800, *p* = .071) (Figure 1d–f).

Notably, we further conducted a subgroup analysis encompassing participants with limited educational attainment (< 9 years of education) to elucidate the efficacy of VCAT within this demographic. The findings revealed that, in discerning cognitive impairment (MCI + mild dementia) from cognitively healthy individuals (HC), VCAT demonstrated markedly superior performance (AUC (95% CI): 0.958 (0.916–1.000)) compared to both MMSE (AUC (95% CI): 0.929 (0.825–1.000)) and MoCA (AUC (95% CI): 0.934 (0.878–0.990)). However, akin to the results observed in the entire cohort, VCAT exhibited marginally diminished diagnostic efficacy relative to the other two scores when distinguishing mild dementia from nondemented groups (HC + MCI) (Table S2).

## 4 DISCUSSION

Given that cognitive abilities are influenced by cultural factors, assessments focusing on these may harbor cultural biases, emphasizing the significance of a shared cultural identity based on common national origins, traditions, literacy (or educational attainment), and language, collectively termed as ethnicity (Rosselli et al., 2019). Consequently, there arises a necessity for a test that remains impartial to language and cultural nuances. The VCAT, a language‐neutral visual‐based cognitive assessment test, exhibits promise in identifying early cognitive impairment within multilingual and multicultural communities (Lim et al., 2018). However, its validation is lacking in China, the most populous nation globally. Our current study demonstrates the VCAT's exceptional discriminative capability for screening cognitive impairment (AUC = 0.929, 95% CI 0.872–0.966), aligning closely with the performance of MoCA and MMSE within the Chinese population. Our results resonate with a prior study encompassing diverse ethnicities (78.1% Chinese, 12.3% Indian, 2.7% Malay, and 6.8% others), reporting a similar diagnostic efficacy with an AUC of 0.933 (Kandiah et al., 2016). Furthermore, our investigation underscores the VCAT's commendable performance in screening mild dementia, boasting a high AUC (0.940), sensitivity (90.9%), and specificity (86.1%), comparable to MoCA's AUC of 0.902. Consequently, the VCAT exhibits significant potential for adoption as a cognitive screening tool across the broader Chinese population.

The VCAT exhibits commendable convergent and discriminant validity (Cronbach & Meehl, 1955). Convergent validity assesses if the selected items adequately represent the subject or content under examination. In our investigation, Spearman's correlation coefficients between the scale items and the total score ranged from 0.390 to 0.898, with all analyses revealing significant correlations (all *p* < .01). Discriminant validity was evident in the domains of episodic memory, visuospatial, and language, displaying stronger correlations with their respective NPA domains than with other NPA domains. The correlation between attention and Clock Drawing Test ranks stood out as the highest among NPA subdomains, likely owing to the Clock Drawing Test's psychometric properties, measuring attention, executive function, and visuospatial skills (Henderson et al., 2007). Similarly, the correlation between the executive function of VCAT and the Boston Naming Test surpassed that of other NPA subdomains. This could be attributed to the Boston Naming Test evaluating not only language function but also identification ability, significantly influenced by executive function (Whiteside et al., 2016). Groups of different diseases with distinct psychological profiles, such as Alzheimer's disease patients manifesting primarily in memory decline, vascular cognitive impairment patients displaying a decline in executive function, and semantic dementia patients exhibiting impairments in semantic knowledge (Bang et al., 2015; Rundek et al., 2022), could be distinguished by a vast majority of the subscales and the total scores. Criterion‐related validity, also termed criterion validity (Scanlon et al., 2016), was confirmed through positive correlations between the VCAT total score and those of the widely utilized scalar effect scales (MMSE and MoCA), both rigorously evaluated in China, with correlation coefficients of 0.785 and 0.795 (all *p* < .01). Internal consistency reflects the homogeneity among the various items of a scale, commonly assessed using Cronbach's α coefficient, where a higher coefficient indicates greater consistency in measuring the screening intention across different items (Souza et al., 2017). The relatively lower Cronbach's alpha (.577) coefficient in VCAT may be attributed to inconsistencies in the degree of impairment across various cognitive domains among patients with different types of cognitive disorders.

In our study cohort, the mean duration for VCAT administration spans from 12 to 16 min, aligning closely with the time required for MMSE or MoCA, reported to be around 10 min (Zhuang et al., 2021). This timeframe is shorter than the previously documented duration of 15.7 ± 7.3 min (Kandiah et al., 2016), possibly attributed to variations in the language of test instructions and disparities in the proficiency levels of the study population. The VCAT, with the majority of the research recruiting patients from memory clinics and elderly centers, is accompanied by a standardized set of instructions, primarily administered by neurologists who have undergone training to ensure a standardized process. Evaluator training is uncomplicated due to the lucidity and interpretability of the scale's instructions and scoring system (refer to Supplemental Material 1–3), facilitating widespread adoption. Although patients with severe cognitive impairments (such as advanced dementia or those with agitated psychiatric symptoms) or obvious orientation disturbance may not fully cooperate with the scoring, making assessment challenging, the image‐based cognitive screening proves to be engaging for most patients with mild cognitive impairments. Its lack of reliance on text makes it more accessible and appealing, providing a degree of entertainment in the evaluation process.

The VCAT threshold for detecting mild dementia was established at 15, a value below the benchmarks set by prior validation and research endeavors, which recorded a score of 20 (Kandiah et al., 2016; Low et al., 2020). Interestingly, our study designated 20 as the cutoff point for identifying cognitive impairment, a figure lower than the reported thresholds of 22 or 24 in preceding investigations (Kandiah et al., 2016; Low et al., 2020). We attribute this discovery to the comparatively modest sample size within the dementia subgroup, manifesting an average inferior cognitive function possibly due to a lack of early‐stage disease awareness in our study, in contrast to earlier investigations. Furthermore, it may also be linked to disparities in subject characteristics between our cohort and previous studies, notably in terms of age levels.

The level of education has often been shown to influence the performance of cognitive tests, such as the MMSE and MoCA (Lee et al., 2008; Wong et al., 2015). Remarkably, we observed that the impact of education on VCAT score in the current study is less pronounced than its influence on MMSE and MoCA, aligning with the conclusions drawn in a prior investigation (Kandiah et al., 2016). Subgroup analysis indicates the efficacy of VCAT scores remains robust within the cohort of individuals with limited educational attainment (< 9 years of education), particularly in distinguishing cognitive impairment (MCI + mild dementia) from HC. This implies the potential applicability of VCAT among those with lower educational backgrounds.

Concerning the impact of age on cognitive test performance, it was postulated that auditory memory scores might decline due to age‐related hearing impairment. Given the VCAT's reliance on visual‐based memory assessment rather than auditory, we have demonstrated its effectiveness in detecting cognitive impairment with heightened sensitivity and specificity (Garami et al., 2020; Sarazin et al., 2007). Consequently, the expectation was for VCAT to exhibit superior performance concerning age‐related influences compared to other scales. The correlation observed between age and scores across all participants may find its roots in the younger participants possessing a more extensive knowledge of new concepts requiring recognition during the test in our study.

Episodic memory deficits have been identified as predictors of progression to Alzheimer's disease (AD) among those with MCI (Grober et al., 1988; Rabin et al., 2012; Sarazin et al., 2007; Tounsi et al., 1999). Consequently, delayed episodic memory recall emerges as a highly sensitive predictor of AD (Garami et al., 2020). Conversely, executive function is an early cognitive impairment marker in patients with vascular dementia. In light of VCAT's specific emphasis on episodic memory and executive function, there is a possibility that it holds unique advantages in distinguishing between AD and vascular dementia. Further research is necessary to substantiate this potential.

Our study faced several limitations. First, the sample size was relatively modest, necessitating validation through more expansive investigations. Second, the intrinsic language‐neutral attributes of the test items and the visually‐oriented nature of the assessment may diminish the VCAT's validity in cases involving substantial visual impairment. Thirdly, the applicability of the VCAT in diagnosing atypical dementias, including frontotemporal dementia, posterior cortical atrophy of the AD variant, and primary progressive aphasia, requires further elucidation. Fourthly, prevalent ophthalmic issues among the elderly demographic pose potential confounding variables.

There were several limitations in our study. First, the sample size was relatively modest, necessitating validation through more expansive investigations. Second, the applicability of the VCAT in diagnosing atypical dementias, including frontotemporal dementia, posterior cortical atrophy of the AD variant, and primary progressive aphasia, requires further elucidation. Thirdly, ophthalmic issues, prevalent in the elderly demographic, may act as confounding variables. Nonetheless, our study systematically excluded individuals manifesting evident visual impairment, potentially mitigating the confounding impact. Finally, the study's participant cohort was delimited to individuals seeking care at specialized cognitive impairment clinics, given the prevalent use of cognitive screening tools within this demographic. Therefore, caution should be exercised when extrapolating these results to the general population. Further community or population‐based clinical researches are warranted to validate the application of VCAT in the broader population. Nonetheless, our study indicates that within cognitive impairment clinics, VCAT remains an excellent tool, not inferior to conventional cognitive screening scales.

## 5 CONCLUSION

The VCAT scoring system demonstrated commensurate efficacy with the MoCA in discerning individuals with MCI from HCs in Chongqing, China. Moreover, it exhibited similar discriminatory prowess to the MMSE in pinpointing cases of mild dementia within the HC cohort. Notably, the VCAT score system, originating from Singapore with an English language background, can be easily utilized in a Chinese cultural context. There is potential for it to serve as a simple and widely applicable scoring tool in Chinese community settings. Further research is imperative to ascertain its generalizability and effectiveness across diverse populations.

## AUTHOR CONTRIBUTIONS

**Yidan Liu**: Conceptualization; formal analysis; data curation; writing—original draft;Methodology; software. **Binbin Xie**: date curation. **Qin Li**: date curation. **Shufang Xiao**: data curation. **Jiamin Li**: Data curation. **Nagaendran Kandiah**: Writing—review and editing. **Kok Pin Ng**: Writing—review and editing. **Liumi Jiang**: Conceptualization; funding acquisition; writing—review and editing. **Xiaofeng Li**: Conceptualization; funding acquisition; writing—review and editing; supervision.

## CONFLICT OF INTEREST STATEMENT

There are no competing interests to disclose.

### PEER REVIEW

The peer review history for this article is available at https://publons.com/publon/10.1002/brb3.3413.

## Supporting information

Supplemental Material

## Data Availability

The data are available upon reasonable request from the corresponding author.
