# Supplementary material for: Visual Cognitive Assessment Test: Utility of the brief cognitive battery for early screening of cognitive impairment in Chongqing, China
Source: Brain Behav. 2024 Feb 5;14(2):e3413. doi: 10.1002/brb3.3413 (PMC10839535; doi:10.1002/brb3.3413)
Supplement: Supplementary file 1 — Supplemental Material [file BRB3-14-e3413-s001.docx]

**Supplemental Material 1. Patient Answer Sheet of Visual Cognitive Assessment Test**

| **Language administered: English / Mandarin / Malay / Hokkien/ Teochew / Cantonese / Others (Please specify):**  **Time Start:** | |
| --- | --- |
| **Memory**   1. Scenario   Please look at the picture below and (1 min):   - - Name the location   - Name me the items that you can see   Dog Coconut Tree Kite Crab Lady Bone |  |
| **Visuospatial**  2)  Which of the following option (A, B, C or D) when folded up will result in the figure below? Please circle one option.  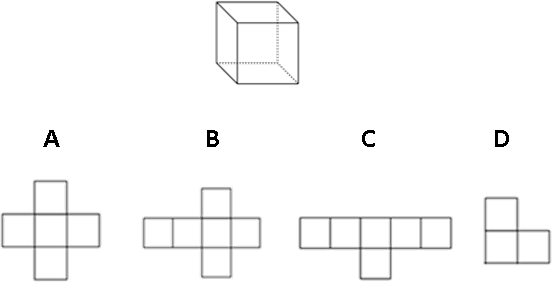 | **/ 1** |
| 3) Grid  Please copy the figure from on the left to the empty one on the right as fast as you can.  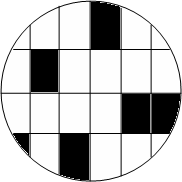 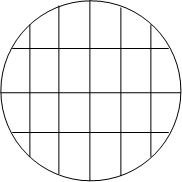 | **/ 2** |

| **Attention/ Working Memory**  4) Shape Cancellation  Cancel the following shapes: and . You have 1 minute.  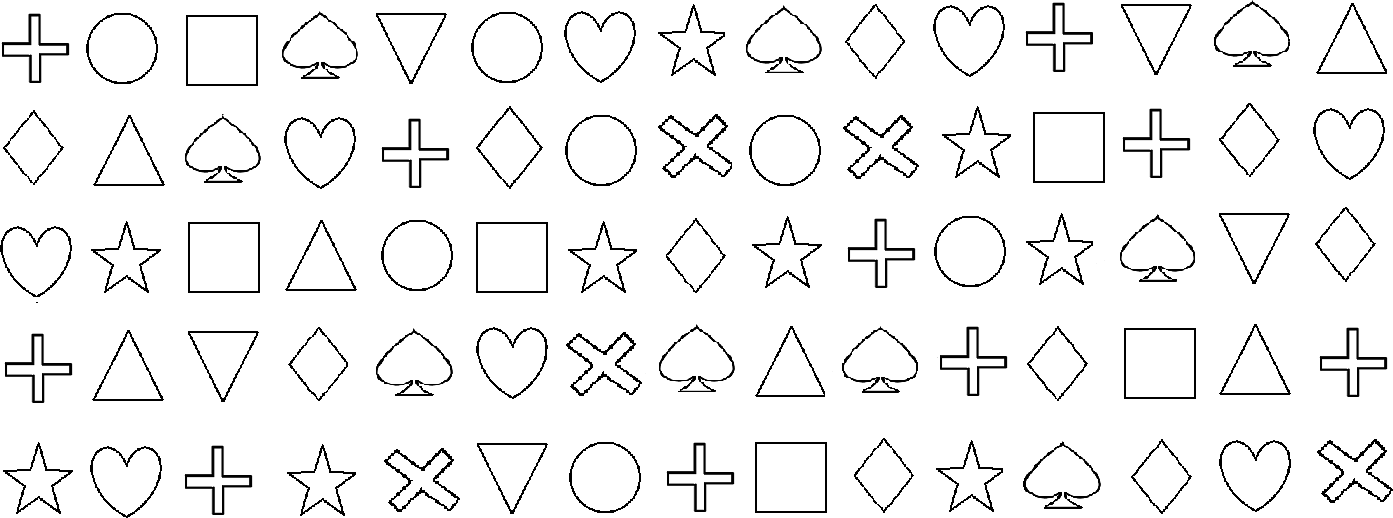  Time: | **/ 3** |
| --- | --- |
| **5) Delayed Recall: Scenario**  You were shown a picture of a scenario earlier. There are **3** objects below that were **NOT** present in the picture earlier. Please circle these three items.  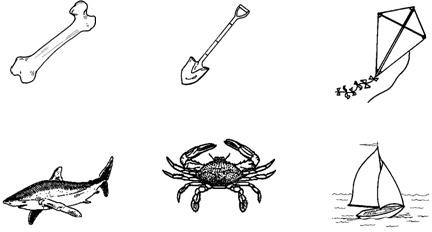 | **/3** |
| **Memory**  6) Shapes  Please look at the shapes and try to remember as many elements as you can. I will ask you about it later (10s). |  |


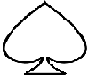

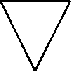


| **Language**  7) Naming  Please name the items below.  Paperclip Bicycle Lightbulb | | | | | | | | | **/3** |
| --- | --- | --- | --- | --- | --- | --- | --- | --- | --- |
| 8) Semantic Fluency  Please name as many vegetables as you can. | | | | | | | | | **/ 2** |
|  | Vegetables | | | | | | Total |  |  |
|  |  | 1-15s | 16s-30s | 31s-45s | 46s-60s |  |  |  |  |
| **Executive Function**  9) Gears   1. If Gear 1 is turning in the indicated direction, please draw the arrow in which will Gear **2** turn.   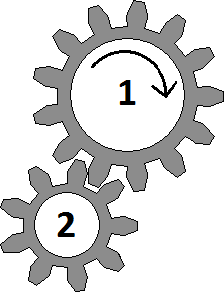   1. If Gear 1 turns in the indicated direction, in which direction will Gear **3** turn?   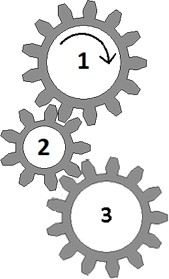 | | | | | | | | | **/ 3** |

| 10) Delayed Recall: Shapes  I showed you some shapes earlier. Please try to recall and fill in the boxes below with the shapes you saw.  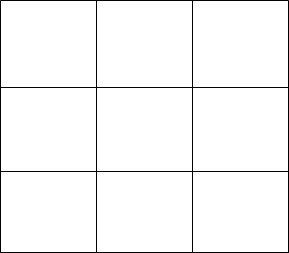 | **/ 2** |
| --- | --- |
| **Memory**  11) Objects  Please name the objects. * Repeat this twice. I’d like you to remember these FOUR objects, I will ask you about them later (20s). |  |
| **Executive Function**  12) Patterns  Take a look at the patterns below and fill in the empty boxes with the correct patterns.  (a) (  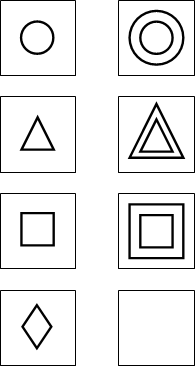 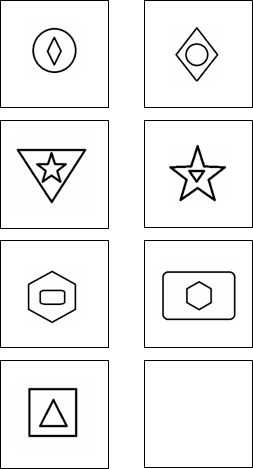 | **/ 2** |

b)

| 13) Category  Which of the following options (A, B or C) is the best option to place inside the empty box? Please circle your option.  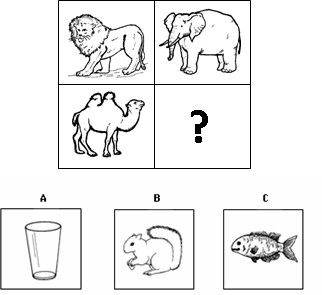  **Please turn over.** | **/ 1** |
| --- | --- |

| **Delayed Memory: Objects**  14) I showed you four objects earlier. Can you recall what the four objects are? | **/8** |
| --- | --- |
| **Time End:** |  |

|  | Hamburger | Bench | Broom | Teapot | Score |
| --- | --- | --- | --- | --- | --- |
| Uncued (2 points) |  |  |  |  |  |
| Cued (1 point) |  |  |  |  |  |

|  |  |  |  |  |  |
| --- | --- | --- | --- | --- | --- |
|  |  |  |  |  |  |
|  |  |  |  |  |  |

**Supplemental Material 2. Instructions for the Scoring System of the Visual Cognitive Assessment Test**

| **VCATS TEST INSTRUCTIONS** | | | |
| --- | --- | --- | --- |
|  | Test | Instructions | Scoring System |
| 1) | Memory: *Scenario* | Please look at the picture below **(1 min)** and:   - Name the location - Name all the items that you see   Try to remember all the items that you see, I will ask you about it later. | NO POINTS GIVEN:   - Location: Beach, Seaside (If participant cannot name location, DO NOT continue with the test) - Items: Dog, Coconut Tree, Kite, Crab, Lady & Bone (If participant cannot name all the items, DO NOT continue with the test) |
| 2) | Visuospatial: *Cube* | Which of the following option (A, B, C or D) when folded up will result in this figure? (Point to the  cube) Circle the correct answer | Incorrect (A, C & D) = 0 Correct (B) = 1  Answer: B |
| 3) | Visuospatial: *Grid* | Please take a look at this figure and copy it on the empty one on the right. Please go as fast as you can. | **In 30s,**  0 – 3 correct boxes = 0 point  4 – 5 correct boxes = 1 point  6 (All) correct boxes = 2 points  * If participant finishes it after 30s = 0 point |
| 4) | *Attention/ WM* | There are some shapes, I’d like you to take a look at the shapes. From left to right, one row at a time, I’d like you to cancel these 2 shapes (point to the spade and the downward triangle).  You have 1 minute to complete it.  * If the participant finishes in less than 1 minute, do not stop them. | 0 -1 error = 3 points  2 errors = 1 point  3 or more errors = 0 point |
| 5) | Delayed Recall:  *Scenario* | You were shown a picture of a scenario earlier. There are **THREE** objects below that were **NOT** present in the picture earlier. Please circle the three  items. | Answer: Shark, Spade and Boat  1 point for EACH correct answer |

| 6) | Memory: *Shapes* | Please look at the shapes and try to remember as many elements as you can. I will ask you about it  later. **(10s)** | NO POINTS GIVEN |
| --- | --- | --- | --- |
| 7) | Language: *Naming* | Please name the items below.  * If they ask you if they need to remember these items, tell them don’t need to. | - Paperclip, Clip - Bicycle, Bike - Lightbulb, Bulb (Anything that has the word bulb) 1 point for EACH correct answer |
| 8) | Language: *Semantic Fluency* | Please name me as many vegetables as you can. I will tell you when to stop.  **TIME: 1 MIN** | - 8 – 10 words: 1 point - 11 or more words: 2 points |

| 9) | Executive Function:  *Gears* | 1. If Gear 1 is turning in this direction (POINT TO Gear 1), please draw the arrow in which **Gear 2** will turn in. 2. If Gear 1 is turning in this direction (Point to Gear 1), please draw the arrow in which **Gear 3** will turn in.   * If participant does not get it right, do not correct them. | Both gears wrong = 0 point Either 1 gear correct = 1 point 2 gears correct = 3 points  a)  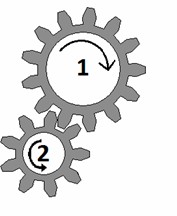  b)  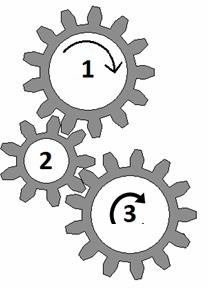 |
| --- | --- | --- | --- |

|  |  |  |  |
| --- | --- | --- | --- |
| 9) | Delayed Recall:  *Shapes* | I showed you some shapes earlier. Please try to recall and fill in the boxes with the shapes. | 0 – 1 shape correct = 0 point  2 – 3 correct = 1 point  4 (All) correct = 2 points  *** BOTH SHAPE and POSITION MUST BE CORRECT**  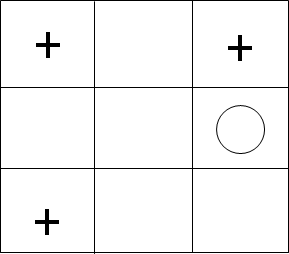 |
| 10) | Memory: *Objects* | Please name the objects. REPEAT TWICE. Now, please try to remember these four items, I will ask you about them later. (**20s**) | NO POINTS GIVEN |
| 11) | Executive Function:  *Patterns* | Please take a look at the patterns below and fill in the empty boxes with the correct patterns.  * Do 1 at a time, can point to direct attention to the 1^st^ pattern. | 1 point for EACH correct answer.  SIZE does not matter, as long as you can make out that it is roughly the correct answer   1. b)   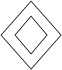 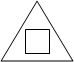 |
| 12) | Executive Function:  *Category* | Which of the following options (A, B, C or D) is the best option to place inside the empty box? Please  circle your choice | 1 point for correct answer Answer: B |

| 13) | Delayed Recall:  *Objects* | I showed you 4 objects earlier. Can you remember what they were?  * If participant unable to answer, provide cues | Correctly UNCUED: 2 points  Correctly CUED: 1 point Incorrect even with cues: 0 point |
| --- | --- | --- | --- |

**Supplemental Material 3. Rater's stimulus booklet of the Visual Cognitive Assessment Test**

**Memory: *Scenario***

Please look at the picture below (1 min) and:

- Name the location
- Name all the items that you see

Try to remember all the items that you see, I will ask you about it later.
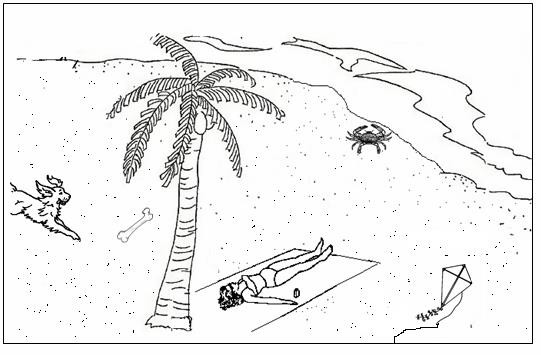


**Memory: Shapes**

Please look at the shapes and try to remember as many elements as you can. I will ask you about it later.


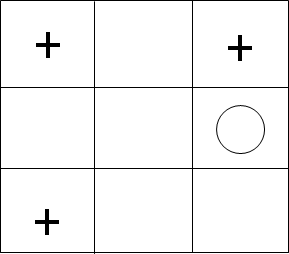


**Language: Semantic Fluency**

Please name me as many vegetables as you can. I will tell you when to stop.

TIME: 1 min


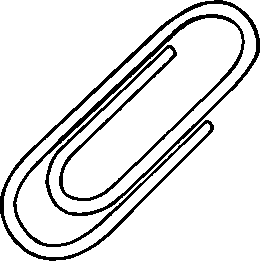

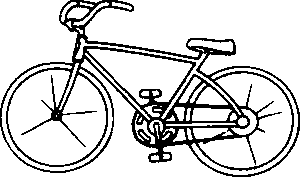

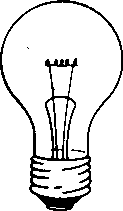


**Memory: Objects**


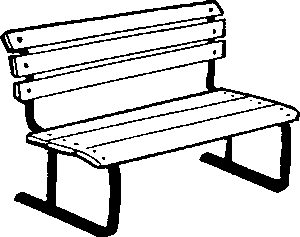
Please name the objects. REPEAT TWICE. Now, please try to remember these four items, I will ask you about them later.

TIME: 20s


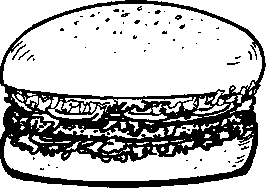


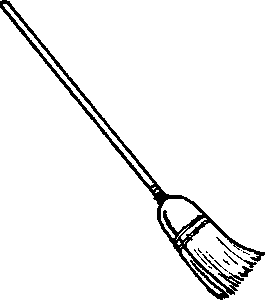

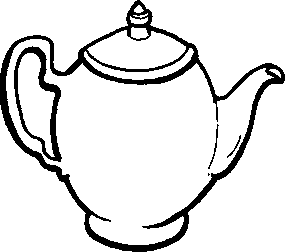


**Delayed Recall:**

I showed you 4 objects earlier. Can you remember what they were?

* If participant unable to answer, provide cues


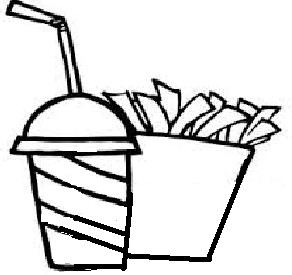


**
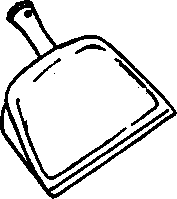
**

**
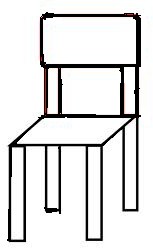
**

**
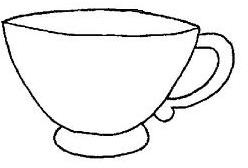
**

**Supplemental Table 1. Multivariate linear regression analyses.**

|  | **VCAT** | | **MMSE** | | **MoCA** | |
| --- | --- | --- | --- | --- | --- | --- |
|  | **β (95%CI)** | **P value** | **β (95%CI)** | **P value** | **β (95%CI)** | **P value** |
| **Age (year)** | -0.15 (-0.30−-0.01­) | 0.034 | -0.09 (-0.20−0.02) | 0.104 | -0.11 (-0.23−-0.00) | 0.041 |
| **Sex (male)** | -0.05 (-2.33−2.22) | 0.963 | -1.06 (-2.85−0.71) | 0.238 | -1.00 (-2.85−0.85) | 0.285 |
| **Education (years)** | 0.29 (0.03−0.54) | 0.026 | 0.34 (0.15−0.55) | <0.001 | 0.48 (0.28−0.69) | <0.001 |

Abbreviations: MMSE, Mini Mental State Examination; VCAT, Visual Cognitive Assessment Test; MoCA, Montreal Cognitive Assessment

**Supplemental Table 2. Receiver operating characteristic (ROC) analysis of the MMSE, the MOCA, and the VCAT for low-education participants**

|  | **Index** | **ROC area under the curve** | **95% confidence interval** | **Cut-off point** | **Sensitivity** (**%**) | **Specificity** (**%**) |
| --- | --- | --- | --- | --- | --- | --- |
| HCs *vs.* MCI +mild dementia | MMSE | 0.929 | 0.825−1.000 | 25.5 | 0.881 | 0.810 |
|  | MoCA | 0.934 | 0.878−0.990 | 18.5 | 0.833 | 0.952 |
|  | VCAT | 0.958 | 0.916−1.000 | 19.5 | 0.857 | 0.905 |
|  | MMSE+ MoCA | 0.940 | 0.887−0.993 | − |  |  |
|  | MMSE+ VCAT | 0.962 | 0.922−1.000 | − |  |  |
| HCs +MCI *vs.* mild dementia | MMSE | 0.964 | 0.918−1.000 | 20.5 | 0.944 | 0.867 |
|  | MoCA | 0.902 | 0.828−0.975 | 14.5 | 0.833 | 0.844 |
|  | VCAT | 0.912 | 0.839−0.986 | 14.5 | 0.944 | 0.800 |
|  | MMSE+ MoCA | 0.967 | 0.916−1.000 | − |  |  |
|  | MMSE+ VCAT | 0.964 | 0.916−1.000 | − |  |  |
| MCI *vs.* mild dementia | MMSE | 0.939 | 0.863−1.000 | 18.5 | 0.778 | 0.958 |
|  | MoCA | 0.819 | 0.693−0.946 | 14.5 | 0.833 | 0.708 |
|  | VCAT | 0.841 | 0.717−0.966 | 13.5 | 0.833 | 0.750 |
|  | MMSE+ MoCA | 0.944 | 0.860−1.000 | − |  |  |
|  | MMSE+ VCAT | 0.939 | 0.862−1.000 | − |  |  |

Abbreviations: HCs, healthy controls; MCI, mild cognitive impairment; MMSE, Mini Mental State Examination; VCAT, Visual Cognitive Assessment Test; MoCA, Montreal Cognitive Assessment.
